# No significant role for beta tubulin mutations and mismatch repair defects in ovarian cancer resistance to paclitaxel/cisplatin

**DOI:** 10.1186/1471-2407-5-101

**Published:** 2005-08-11

**Authors:** Bárbara Mesquita, Isabel Veiga, Deolinda Pereira, Ana Tavares, Isabel M Pinto, Carla Pinto, Manuel R Teixeira, Sérgio Castedo

**Affiliations:** 1 Department of Genetics, Portuguese Oncology Institute, 4200-072 Porto, Portugal; 2 Department of Medical Oncology, Portuguese Oncology Institute, 4200-072 Porto, Portugal; 3 Department of Pathology, Portuguese Oncology Institute, 4200-072 Porto, Portugal

## Abstract

**Background:**

The mechanisms of chemoresistance in ovarian cancer patients remain largely to be elucidated. Paclitaxel/cisplatin combination is the standard chemotherapeutic treatment for this disease, although some patients do not respond to therapy. Our goals were to investigate whether *TUBB *mutations and mismatch repair defects underlie paclitaxel and cisplatin resistance.

**Methods:**

Thirty-four patients with primary ovarian carcinomas (26 serous and eight clear cell carcinomas) treated with paclitaxel/cisplatin were analysed. *TUBB *exon 4 was analysed by nested PCR after a first round PCR using intronic primers. Microsatellite analysis was performed with the quasimonomorphic markers BAT 26 and BAT 34.

**Results:**

Twenty-two of the 34 ovarian cancers (64.7%) presented residual tumour after surgery, seven of which (7/22; 31.8%) were shown to be chemoresistant (five serous and two clear cell tumours). Sequence analysis did not find any mutation in *TUBB *exon 4. Microsatellite instability was not detected in any of the ovarian carcinomas.

**Conclusion:**

We conclude that *TUBB *exon 4 mutations and mismatch repair defects do not play a significant role in paclitaxel/cisplatin resistance.

## Background

Ovarian cancer is the fourth most common cancer in women [1]. The standard treatment for ovarian cancer is cytoreductive surgery followed by combination systemic chemotherapy [2]. Since the middle 90's, the combination paclitaxel/cisplatin became the standard chemotherapeutic treatment for poor prognosis ovarian cancer [3]. Nevertheless, some patients are resistant to this chemotherapeutic treatment, making it important to clarify the underlying mechanisms of resistance [4].

Paclitaxel binds to microtubules and causes kinetic suppression (stabilisation) of microtubule dynamics, promoting their polymerisation and cell cycle arrest in mitosis (antimitotic activity), which probably leads to apoptosis [5,6]. Microtubules are composed of a dimeric protein, tubulin, with alpha (α) and beta (β) tubulin heterodimers in dynamic equilibrium. The β-tubulin gene (*TUBB*), mapped to 6p21.3 [7], is composed of four exons and encodes a 445-aminoacid protein with GTPase function to which paclitaxel preferentially binds [8]. Cisplatin is activated intracellularly and establishes inter- and intra-strand DNA adducts that block replication and translation. The fate of cells after cisplatin exposure depends both on the extent of DNA damage and the cellular response to it, and apoptosis can be induced as a consequence [5,9]. Although the specific mechanism that triggers apoptosis is not totally clear, some evidence suggests that this process can be mediated by the DNA mismatch repair system (MMR) [5,9].

Drug resistance is considered a multifactorial process, but the detailed mechanisms are still unknown. Recently, point mutations in the β-tubulin gene, predominantly in exon 4, were associated with resistance to paclitaxel [10-12]. Resistance to cisplatin was linked with anomalies in the DNA MMR system resulting in microsatellite instability (MSI) [13-16]. In order to evaluate the relevance of these mechanisms to ovarian cancer chemoresistance, we screened *TUBB *exon 4 for mutations and performed MSI analysis in 34 ovarian carcinomas treated with paclitaxel/cisplatin and evaluated patients' response to chemotherapy.

## Methods

### Patient data

Thirty-four primary ovarian carcinoma patients, of serous (26 cases) or clear cell (eight cases) histological types (invasive or borderline), consecutively admitted at the Portuguese Oncology Institute – Porto and treated with the adjuvant chemotherapy scheme paclitaxel/cisplatin, were analysed. Patients previously treated with other chemotherapeutic regimens or radiotherapy were excluded from the study.

Evaluation of treatment responses was done by an oncologist using computerized tomography or magnetic resonance and CA125 quantification, according to international guidelines [17]. Investigators performing laboratory analysis were not aware of chemotherapy response or resistance until the study was completed.

### DNA extraction

Genomic DNA was extracted from chemo-naïve, paraffin embedded tumours, after dissection. Tissue blocks were sectioned, mounted on glass slides, deparaffinised, and stained with haematoxylin and eosin. Tumour areas were identified under the microscope on each slide and marked. Areas with at least 70% of cancer cells were identified on tissue sections. Selected paraffin blocks were sectioned (5μm sections) and mounted in microscope slides. Marked tumour areas were selected with a sterile razor blade. Three sections of tissue were incubated in a solution of 10 mM Tris-HCl buffer (pH 8.0), 2.5 mM MgCl_2_, 50 mM KCl, 0.5% (p/v) Tween 20, and 1 mg/mL proteinase K for 48 hours at 55°C.

### *TUBB *exon 4 sequencing

For polymerase chain reaction (PCR) assays, different sets of oligonucleotides were designed to amplify specific regions of *TUBB *exon 4 that code for the GTP and paclitaxel binding sites. To assure that the amplicon was not a pseudogene, the following intronic primer set was used in the first round PCR: 5'AAG-GAG-ATA-CAT-CCG-AGG-GAA-TT3' and 5'AAG-GTA-TTC-ATG-ATG-CGA3'. After checking for first round PCR product in an agarose gel, a 1:10 dilution was used for nested PCRs with the following primers: set 1, 5'AGA-GAG-CTG-TGA-CTG-CCT-G3' and 5'AAG-GTA-TTC-ATG-ATG-CGA3'; set 2, 5'GCT-CTG-GAA-TGG-GCA-CTC3' and 5'CCG-TAG-GTT-GGT-TGT-GGT-CA3'; set 3, 5'CGG-GGA-TCT-GAA-CCA-CCT-T3' and 5'GAG-TGT-CAC-GGC-CTG-GAG-T3'. The PCR products were separated by electrophoresis in agarose gels stained with ethidium bromide and analysed in a transiluminator. All DNA samples were analysed in an automatic DNA sequencer ABI PRISM 310™ Genetic Analyser. The sequences were compared with the genomic sequence GenBank AF070600.

### MSI evaluation

For microsatellite analysis, tumour DNA was amplified with primers for two quasimonomorphic markers BAT 26 (5'GAG-TGT-CAC-GGC-CTG-GAG-T3'; 5'AAC-CAT-TCA-ACA-TTT-TTA-ACC-C3') and BAT 34 (5'ACC-CTG-GAG-GAT-TTC-ATC-TC3'; 5'AAC-AAA-GCG-AGA-CCC-AGT-CT3') [18-20]. Fragments were analysed in an ABI PRISM 310™ Genetic Analyser.

## Results

Twenty-two of the 34 ovarian cancers (64.7%) presented residual tumour after surgery, seven of which (7/22; 31.8%) were shown to be chemoresistant (five serous and two clear cell tumours) (Table 1).

**Figure 1:**
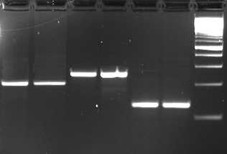
Nested PCR products (in duplicate) obtained with the three primer sets specific for *TUBB *exon 4 in an ovarian carcinoma (from right to left, lane 1: 100 bp step ladder; lanes 2 and 3: 129 bp amplicon – set 1 primers; lanes 4 and 5: 254 bp amplicon – set 2 primers; lanes 6 and 7: 201 bp amplicon – set 3 primers).

**Figure 2:**
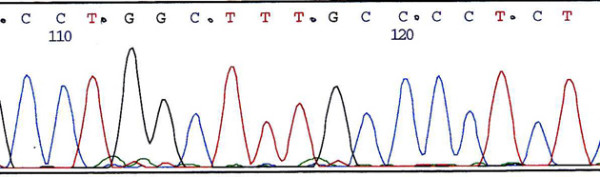
Electrophorogram of part of *TUBB *exon 4 without any sequence variation in an ovarian carcinoma.

Amplicons of 700 bp were observed for all cases after amplification with the intronic primers (data not shown). Nested PCR with primer sets 1–3 specific for *TUBB *exon 4 resulted in amplicons with 129, 254, and 201 bp, respectively (Figure 1). Sequencing analysis of all 34 cases showed no *TUBB *exon 4 mutations (Figure 2).

**Figure 3:**
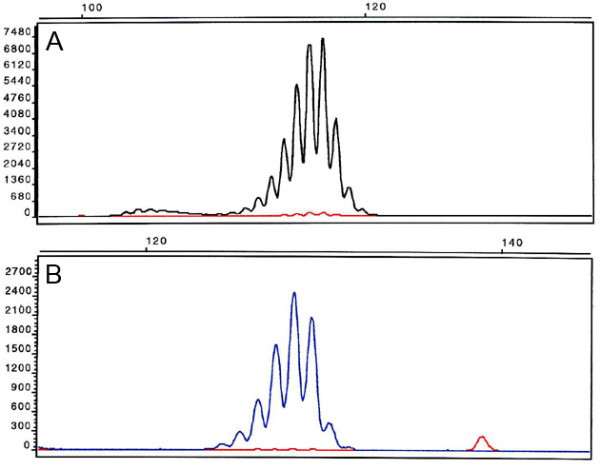
Electrophorograms of BAT 26 (A) and BAT 34 (B) markers in an ovarian carcinoma, showing the same pattern found in normal control DNA.

Microsatellite analysis with markers BAT 26 and BAT 34 (Figure 3) showed the normal pattern in all cases, so no evidence for microsatellite instability was detected in this series.

## Discussion

Tumour resistance to chemotherapy or disease relapses resistant to further treatment after an initial response are common events in current cytotoxic cancer treatment regimens [5]. With regard to ovarian cancer chemotherapy, the current major challenge is to understand why histologically similar tumours behave so differently when treated with the same chemotherapeutic regimen. The action of a drug potentially depends on several mechanisms, namely, metabolisation, access into the tumour microenvironment, intracellular uptake, interaction with the target, and subsequent signalling events [5]. It is therefore important to study the different molecular mechanisms that can be involved in chemotherapy resistance.

The rationale for studying the relationship between *TUBB *gene mutations with paclitaxel resistance came from the studies of Giannakakou *et al *and Gonzalez-Garay *et al *[10,11], who found *TUBB *mutations in ovarian cancer cell lines and in hamster cells, respectively. Subsequently, Monzó *et al *[12] reported *TUBB *mutations in 16 out of 36 (44.4%) paclitaxel resistant tumour samples from patients with advanced non-small cell lung cancer and proposed that *TUBB *mutations could represent a possible mechanism of paclitaxel resistance in that tumour type.

However, our findings in the present study argue against a significant role of *TUBB *gene mutations in paclitaxel resistance in ovarian cancer. In keeping with our results, Sale *et al *[22] and Lamendola *et al *[23] did not detect *TUBB *gene mutations in ovarian cancer samples. Similar findings have recently been obtained for several other tumour types, namely, lung [24], breast [25,26], and gastric cancer [27]. Taken together, our and several other investigations concur that *TUBB *exon 4 mutations are not an important mechanism underlying paclitaxel resistance. To explain the early findings of Monzó *et al *[12], which were not reproduced by other authors, including the present study, Kelley *et al *[24] suggested that the primers used by Monzó *et al *[12] did not allow to discriminate *TUBB *from its pseudogenes [24]. Furthermore, the first studies reporting *TUBB *gene mutations were made in hamster cells [11] and in ovarian cancer cell lines [10] after selection by paclitaxel exposure, something that makes difficult a direct extrapolation of these findings to human tumours.

We have also evaluated the MSI status of the 34 ovarian carcinomas with quasimonomorphic BAT 26 and BAT 34 markers, but did not find any microsatellite unstable tumours. Some authors have described an association between cisplatin resistance and MMR system anomalies in ovarian adenocarcinomas [28-30], as well as in colon cancer cell lines [31-33]. Additionally, other studies found MSI in ovarian cancer, namely, in serous and clear cell histological types [34-40]. However, the frequency of MSI identified in those studies was quite low (0–14.3%), which is in agreement with our findings. To completely rule out any relationship between deficient mismatch repair and cisplatin resistance, one would have to analyse more microsatellite markers in a larger series of tumours paired with normal DNA.

## Conclusion

We conclude that, contrarily to earlier suggestions, *TUBB *exon 4 mutations and MMR defects are not major mechanisms underlying paclitaxel and/or cisplatin resistance in ovarian cancer. Further investigation on alternative mechanisms of resistance to these drugs is warranted. Possible mechanisms to paclitaxel resistance are *P-glycoprotein *overexpression [5,41], differential β-tubulin isotype expression [5,42,43], and apoptosis deregulation [5,44-46]. Decrease in intracellular cisplatin level, increase of tolerance or repair of DNA lesions, and alterations in the apoptotic cascade have also been related with cisplatin resistance [5,47]. These studies are necessary to predict individual response of patients to these chemotherapeutic agents.

## Competing interests

The author(s) declare that they have no competing interests.

## Authors' contributions

BM carried out the molecular genetic studies and drafted the manuscript. IV participated in the design of the study and coordination. DP was responsible for clinical surveillance. AS was responsible for paraffin slides. IMP was responsible for the histopathologic analysis. CP carried out sequence alignment. MT coordinated the study. SC conceived the study and participated in its design and coordination.

## Pre-publication history

The pre-publication history for this paper can be accessed here:



## Figures and Tables

**Table 1:** Clinical, pathological and genetic data of 34 ovarian cancer patients.

***Patient***	***Histological type***	***Stage***	***Grade***	***Residual tumour***	***Treatment response***	***Exon 4 TUBB mutation***	***MSI status***
1	Serous	III	1	> 2 cm	CR	Not present	Stable
2	Serous	III	2	< 2 cm	CR	Not present	Stable
3	Serous	III	3	> 2 cm	CR	Not present	Stable
4	Serous	III	1	> 2 cm	WR	Not present	Stable
5	Serous	III	2	> 2 cm	CR	Not present	Stable
6	Serous	III	2	< 2 cm	CR	Not present	Stable
7	Serous	III	3	> 2 cm	CR	Not present	Stable
8	Serous	III	3	> 2 cm	CR	Not present	Stable
9	Serous	III	3	> 2 cm	CR	Not present	Stable
10	Serous	III	3	> 2 cm	CR	Not present	Stable
11	Serous	III	3	> 2 cm	CR	Not present	Stable
12	Serous	III	3	> 2 cm	WR	Not present	Stable
13	Serous	III	3	> 2 cm	WR	Not present	Stable
14	Serous	IV	3	> 2 cm	PR	Not present	Stable
15	Serous	IV	3	> 2 cm	CR	Not present	Stable
16	Serous	IV	3	> 2 cm	WR	Not present	Stable
17	Serous	IV	3	> 2 cm	WR	Not present	Stable
18	Serous	IV	3	< 2 cm	CR	Not present	Stable
19	Serous	I	1	Absent	-	Not present	Stable
20	Serous	II	3	Absent	-	Not present	Stable
21	Serous	III	1	Absent	-	Not present	Stable
22	Serous	III	3	Absent	-	Not present	Stable
23	Serous	III	1	Absent	-	Not present	Stable
24	Serous	II	B	Absent	-	Not present	Stable
25	Serous	II	B	Absent	-	Not present	Stable
26	Serous	III	B	Absent	-	Not present	Stable
27	Clear cell	I	3	< 2 cm	CR	Not present	Stable
28	Clear cell	II	3	> 2 cm	WR	Not present	Stable
29	Clear cell	III	3	> 2 cm	PR	Not present	Stable
30	Clear cell	IV	3	> 2 cm	WR	Not present	Stable
31	Clear cell	I	3	Absent	-	Not present	Stable
32	Clear cell	I	3	Absent	-	Not present	Stable
33	Clear cell	II	3	Absent	-	Not present	Stable
34	Clear cell	III	3	Absent	-	Not present	Stable

B: borderline tumours; CR: complete response; PR: partial response; WR: without response. The stage classification was according to the *Federation Internationale de Gynecologie *[21] and grading was defined as well differentiated (1), moderately differentiated (2), and poorly differentiated (3).
